# Ultrasonographic Characteristics of Thyroid Nodules with Nondiagnostic and Atypia of Undetermined Significance in Fine-Needle Aspiration Cytology: Correspondence

**DOI:** 10.5334/jbsr.3680

**Published:** 2024-07-19

**Authors:** Ahmet Bozer, Hinpetch Daungsupawong, Viroj Wiwanitkit

**Affiliations:** 1 Department of Radiology, Izmir City Hospital, Laka, 35040 Bayraklı/Izmir, Turkey; 2 Private Academic Consultant, Phonhong, Lao People’s Democratic Republic; 3 Saveetha Medical College and Hospital, Saveetha Institute of Medical and Technical Sciences, Thandaram, Kancheepuram, Chennai, Tamil Nadu, India

## Letter to the Editor,

### Dear editor,

We would like to comment on “Ultrasonographic Characteristics of Thyroid Nodules with Nondiagnostic and Atypia of Undetermined Significance in Fine-Needle Aspiration Cytology [1].” The purpose of this study was to examine the possible consequences of the ultrasonography features of thyroid nodules classified as nondiagnostic (ND) and atypia of undetermined significance (AUS) for clinical care. Numerous patients and nodules were included in the study, and no discernible differences were detected between the ND and AUS categories in any of the nodule characteristics or imaging findings. Both groups showed hyperechoic/isoechoic echogenicity and a preponderance of solid content, along with significant percentages of TI-RADS 4 nodules. All things considered, the study offers insightful information on the ultrasound characteristics of thyroid nodules classified as ND and AUS, information that may be useful in directing clinical judgment calls and patient follow-up plans.

The retrospective form of the study may have limited the ability to control for biases and confounding variables, which is one area where it could have weaknesses. Furthermore, neither the risk of malignancy nor the diagnostic accuracy of ultrasonography characteristics in predicting malignancy for ND and AUS nodules were examined in this investigation. To have a better understanding of the clinical implications of the ultrasound findings in these nodules, it would have been beneficial to add this information. Prospective designs with larger sample sizes and longer follow-up periods may be the focus of future studies to evaluate the diagnostic accuracy of ultrasound features in predicting malignancy for ND and AUS nodules, as well as the risk of malignancy.

There are questions about the logic behind the choice of particular ultrasonography features to analyze, possible flaws in the study design, and the implications of the study’s conclusions for clinical practice. Future studies in this field may examine the use of other imaging modalities, such as contrast-enhanced ultrasonography or elastography, in distinguishing between benign and malignant thyroid nodules classified as ND and AUS. Additionally, research might look into how using ultrasound features affects therapy algorithms and risk assessment models for individuals with these nodules.

## Reply by the Authors,

### Dear authors,

Thank you for your valuable comments on our article titled “Ultrasonographic Characteristics of Thyroid Nodules with Nondiagnostic and Atypia of Undetermined Significance in Fine-Needle Aspiration Cytology [1].” We appreciate your insightful feedback. On behalf of the co-authors, I would like to address the points raised.

We acknowledge that the retrospective nature of our study may have introduced certain limitations, including potential biases and confounding variables. Despite these constraints, we believe our findings contribute significant information to the understanding of the ultrasonographic features of thyroid nodules classified as ND and AUS.

Regarding your concern about the risk of malignancy and the diagnostic accuracy of ultrasound characteristics, we agree that this is a crucial aspect that warrants further exploration. Our primary focus was to describe the ultrasonographic characteristics rather than assess their predictive value for malignancy. Addressing these concerns would indeed require separate studies focusing on the malignancy risk and the predictive value of ultrasound features for these two groups. Future research should consider prospective designs with larger sample sizes and longer follow-up periods to evaluate the diagnostic accuracy of ultrasound features in predicting malignancy for ND and AUS nodules, as well as the overall risk of malignancy.

In our study, ultrasound features and Thyroid Imaging Reporting and Data System (TI-RADS) categories of nodules in both groups were determined based on American College of Radiology criteria. The malignancy rates for each TI-RADS category are well-known: no more than 2% for TR1 and TR2 nodules, 5% for TR3 nodules, 5% to 20% for TR4 nodules, and at least 20% for TR5 nodules [2].

Furthermore, the selection of specific ultrasound features for analysis in our study is based on current literature and the American College of Radiology’s TI-RADS [3]. However, we recognize the potential value of including additional imaging techniques, such as contrast-enhanced ultrasound or elastography, in future research. These techniques can provide a more detailed characterization and aid in effectively distinguishing between benign and malignant nodules. Enhancing the value of easily accessible methods that can be widely disseminated is also necessary.

Lastly, we appreciate your suggestion to investigate how ultrasound features can influence therapeutic algorithms and risk assessment models. Future studies could indeed focus on this aspect to enhance clinical decision-making processes for patients with ND and AUS thyroid nodules.

We are grateful for your constructive feedback, and we believe addressing these points will guide future research in this field. Hopefully, this response adequately clarifies the topic and further highlights the strengths and limitations of our study. Thank you once again for your thoughtful criticism.
